# Supplementary figures and images for: Protease-mediated activation of Par2 elicits calcium waves during zebrafish egg activation and blastomere cleavage
Source: PLoS Biol. 2025 Jun 17;23(6):e3003181. doi: 10.1371/journal.pbio.3003181 (PMC12173237; doi:10.1371/journal.pbio.3003181)

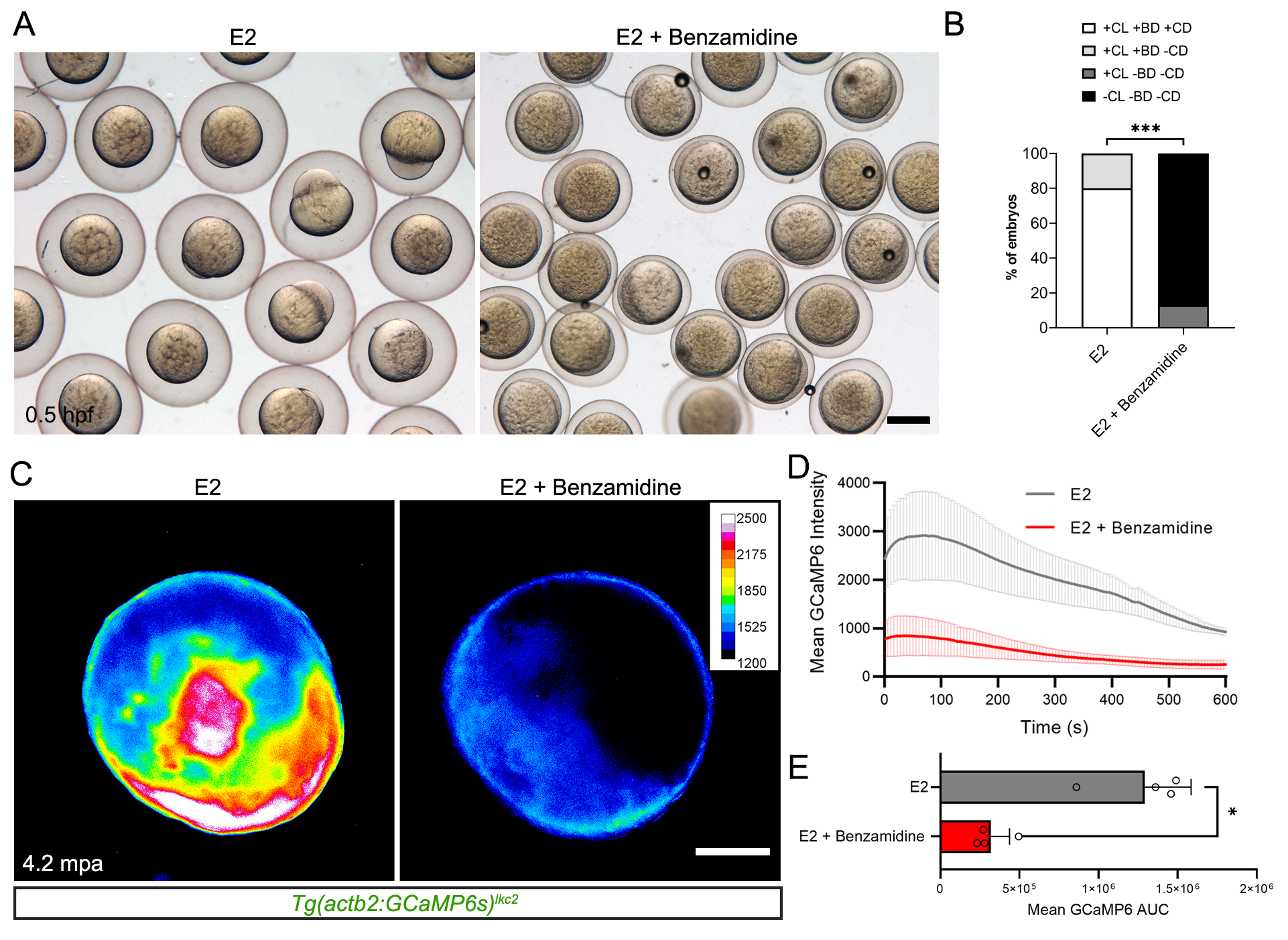

Supplement: S1 Fig — A: Eggs fertilized in vitro in E2 medium with or without 40 mg/ml Benzamidine HCl. B: Proportion of embryos showing egg activation and blastomere division phenotypes for 40 mg/ml Benzamidine HCl treatment vs. E2 alone. Key: CL: Chorion Lift, BD: Blastodisc, CD: Cell Division, +: Present, −: absent. Chi-squared analyses; *** = p < 0.001; n = 100. C: Normalized pseudo-coloured fluorescent images of unfertilized Tg(actb2:GCamP6s)lkc2 eggs indicating Ca2+ dynamics at 250 s during egg activation in E2 or Benzamidine HCl. D: Quantification of changes in mean GCamP6s intensity from fluorescent timelapse of Tg(actb2:GCamP6s)lkc2 eggs activated in E2 (gray line) vs 40 mg/ml Benzamidine HCl treatment (red line) E: Corresponding statistical analyses of GCamP6s intensity AUC from (D). n = 4; Mann–Whitney test; * = p < 0.05. Scale bars: A = 500 µm, C = 200 µm. See file S1 Data for underlying data. (TIF) [file pbio.3003181.s001.tif]

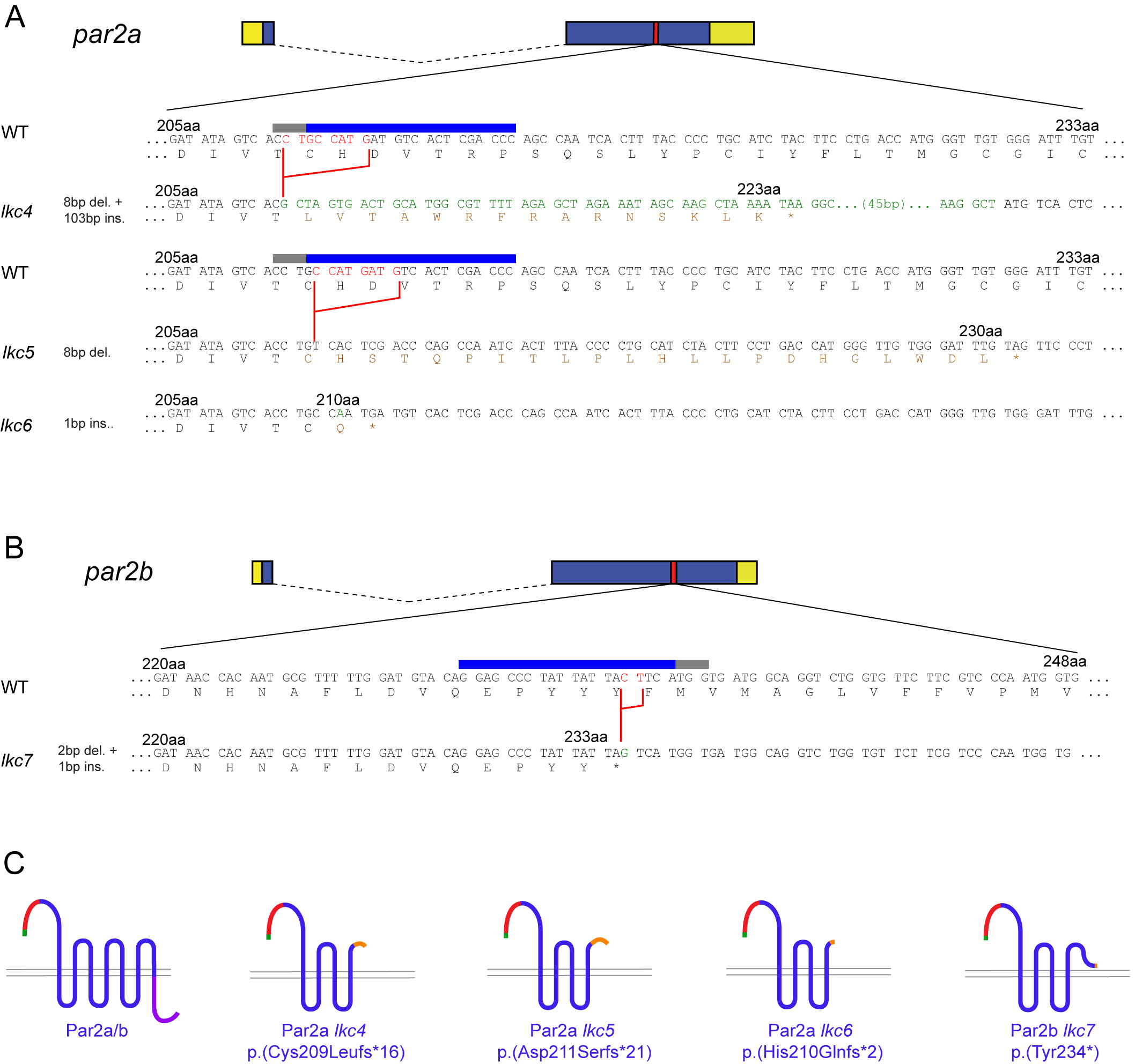

Supplement: S2 Fig — A, B: CRISPR mutagenesis of par2a (A) and par2b (B). Intron-Exon structures are given above. Boxes correspond to exons and dashed lines represent introns. Both genes have 2 exons. Dark blue and yellow boxes represent coding and untranslated regions respectively. Red bars indicate the approximate location of CRISPR target site with the sequence given below for each allele (name and mutation summary given on left), with corresponding WT sequence for comparison. Blue bars designate CRISPR binding sites and gray bars indicate PAM site. Red and green text indicate deleted and inserted nucleotides respectively. Brown amino acid sequences indicate novel amino acids introduced by frameshift. The 103 bp insertion in the par2alkc4 allele shows homology to the Cas9 plasmid template used to synthesize Cas9 RNA. C: Protein schematics for all Par2a and Par2b CRISPR alleles. Green, red, blue, purple, and orange sections indicate signal peptide, tethered inhibition domain, 7-pass transmembrane region, intracellular tail, and induced frameshift regions, respectively. Corresponding allele names and summary given below. (TIF) [file pbio.3003181.s002.tif]

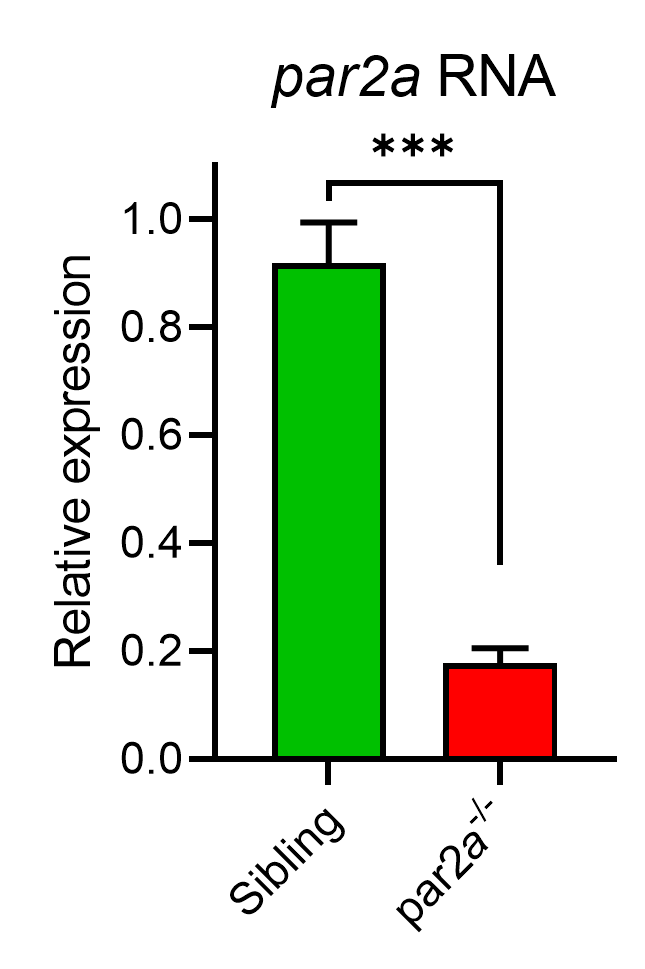

Supplement: S3 Fig — Relative levels of par2a mRNA in 1-cell zygotes derived from par2alkc4/lkc4 homozygous mothers and sibling mothers. Quantitative PCR was performed on cDNA, and par2a transcript levels normalized to eef1a1l1 transcripts. RNA was prepared from pooled zygotes derived from three par2alkc4/lkc4 homozygous mothers and three sibling mothers. All were outcrossed to wild-type fathers. n = 3; unpaired two-tailed t test; *** = p < 0.001. See file S1 Data for underlying data. (TIF) [file pbio.3003181.s003.tif]

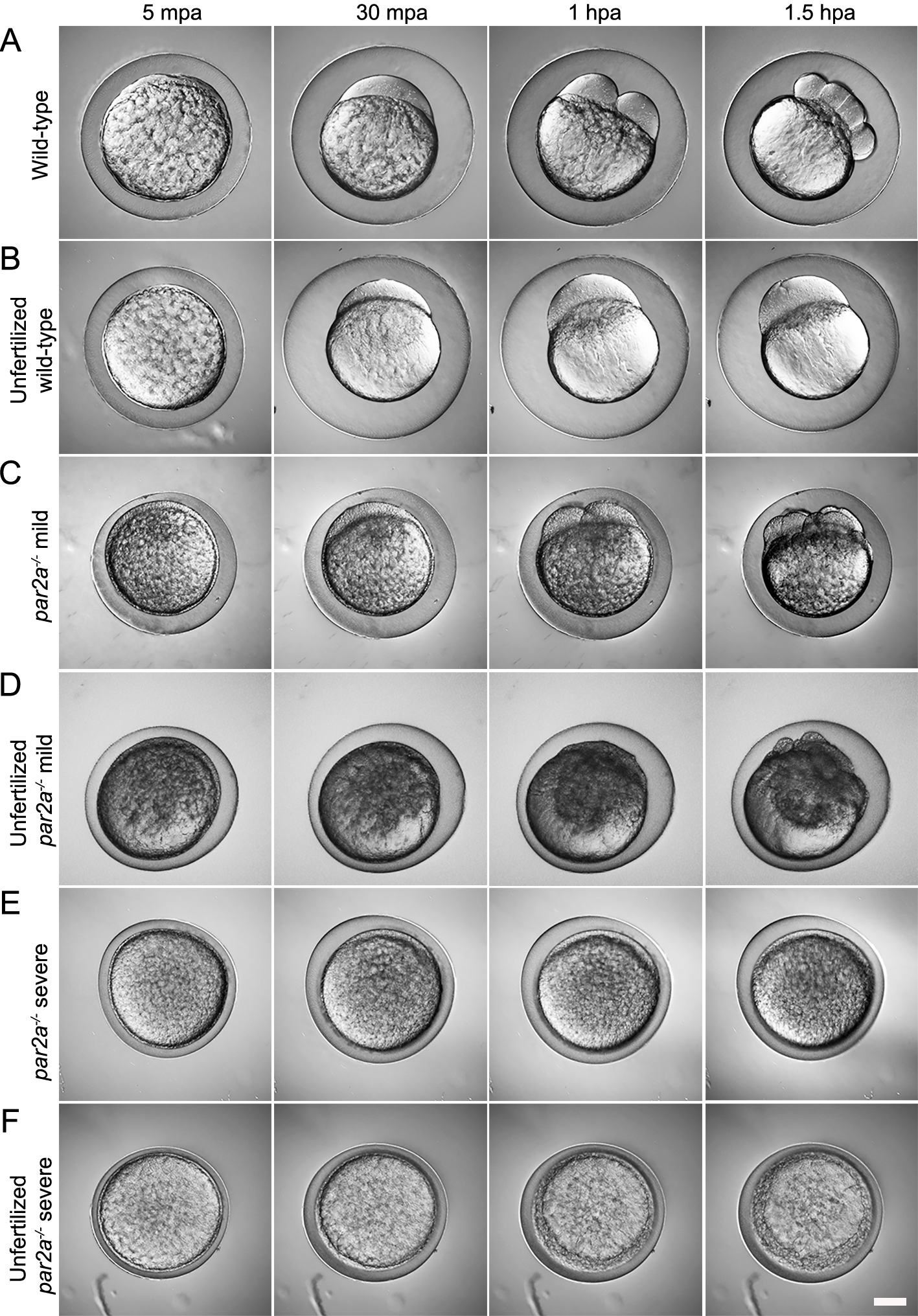

Supplement: S4 Fig — Nomarski images of naturally fertilized (A, C, E) and unfertilized (B, D, F) eggs derived from WT (A, B), mild (C, D), and severe (E, F) par2a mutant females. Eggs were fertilized with WT sperm in A, C, E. Images were taken at 5 min (5 mpa), 30 min (30 mpa), 1 h (1 hpa), and 1.5 h postactivation (1.5 hpa). Chorion elevation is reduced in the par2a mutant derived eggs (C–F) and a defective blastodisc forms only in the mild par2a mutant eggs (C, D), with none forming in either the fertilized or unfertilized severe mutant eggs (E, F). Scale bar: F = 200 µm. (TIF) [file pbio.3003181.s004.tif]

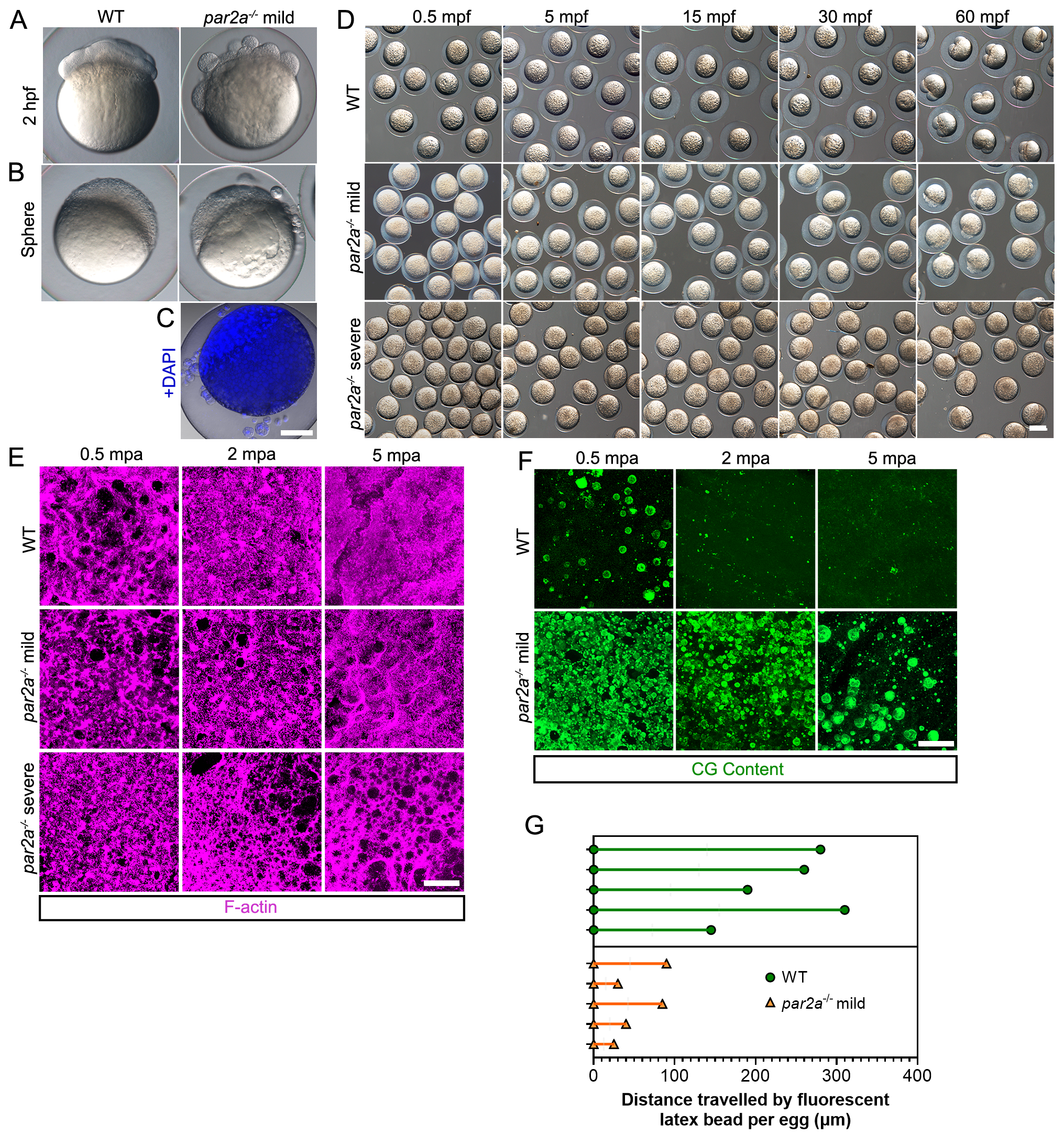

Supplement: S5 Fig — A–C: Nomarski images of WT (left) and mild par2a−/− mutants (right) derived from natural crosses at 2 hpf (A) and sphere stage (4 hpf; B). C: DAPI staining superimposed on Nomarski images of mild par2a−/−- mutants at sphere stage D: Clutches of embryos from WT, mild and severe par2a−/−- mutants generated by IVF and imaged at 0.5, 5, 15, 30, and 60 mpf. E: Projected confocal images showing dynamics of cortical F-actin stained by AlexaFluor-546 Phalloidin at 0.5, 2, and 5 mpa, in E2 activated WT (top), par2a−/−- mild (middle) and severe par2a−/−- mutant eggs (bottom). F: Projected confocal images showing dynamics of Cortical Granule release stained by FITC-MPL at 0.5, 2, and 5 mpa, in E2 activated WT (top), and par2a−/−- mild mutants (bottom) G: Distance traveled by injected fluorescent latex beads in individual fertilized eggs of WT (top; green) vs. mild par2a−/−- mutants (bottom; yellow) from start point (left) to end position at 1 h. Each line represents the distance traveled by tracked beads in each embryo. Scale bars: C = 200 µm; D = 500 µm; E, F = 50 µm. See file S1 Data for underlying data. (TIF) [file pbio.3003181.s005.tif]

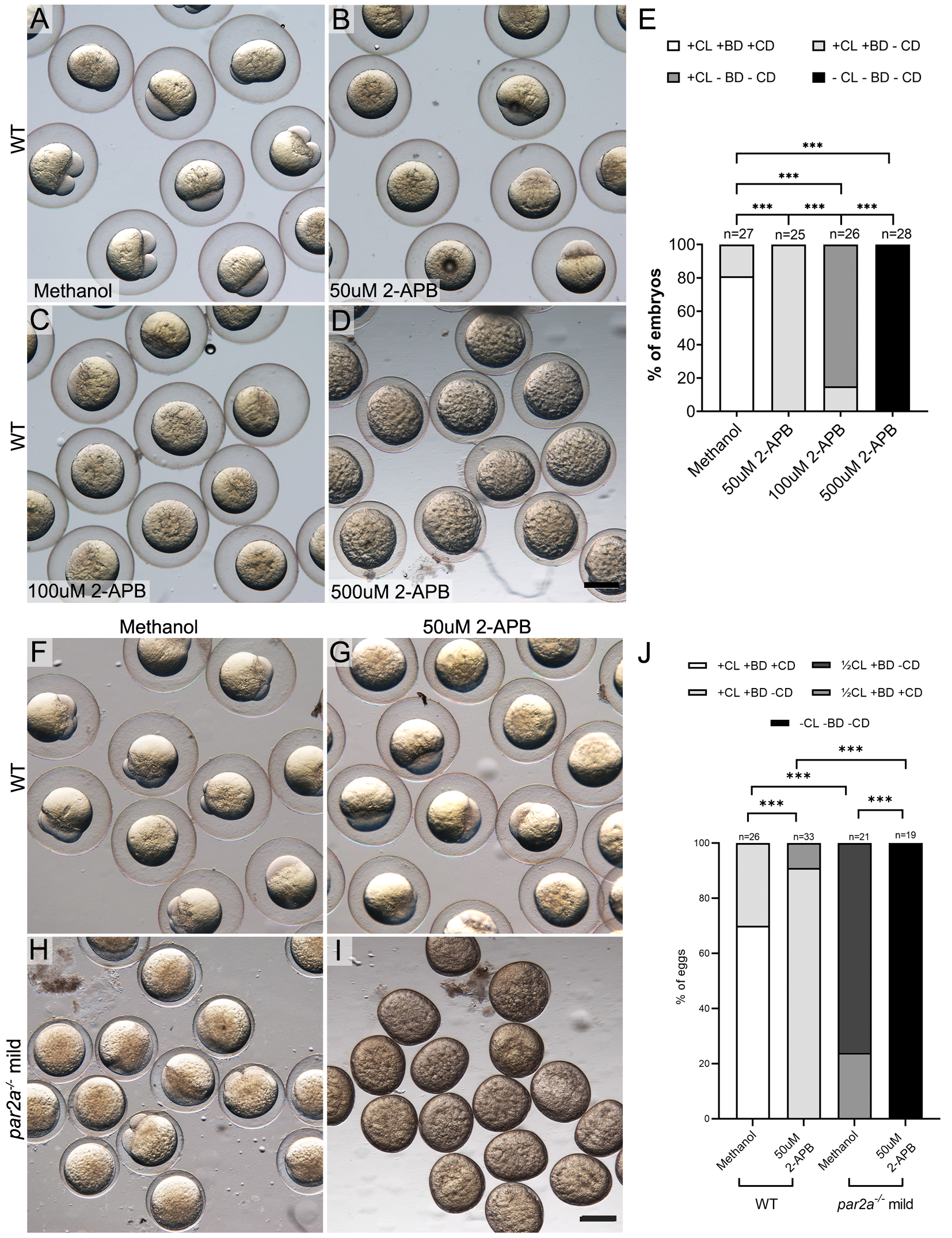

Supplement: S6 Fig — A–D: WT embryos treated with methanol carrier (A), or 50 µM (B), 100 µM (C), and 500 µM (D) of IP3R inhibitor, 2-APB. E: Proportion of egg activation phenotypes presented by different concentrations of 2-APB. Key: CL: Chorion Lift, BD: Blastodisc, CD: Cell Division, +: Present, −: absent. Chi-squared analysis; *** = p < 0.001. F–I: Naturally fertilized WT (F, G) and mild par2a mutant (H, I) embryos treated with methanol (F, H) or a low dose (50 µM) of 2-APB. Low dose of 2-APB strongly exacerbates egg activation defects in mild par2a mutants. J: Counts of proportion of embryos in WT and mild par2a mutants showing extent of phenotype in low dose of 2-APB. Key: CL: Chorion Lift, BD: Blastodisc, CD: Cell Division, +: Present, −: absent, ½: half reduced; Chi-squared analysis; *** = p < 0.001. Scale bars: D, I = 500 µm. See file S1 Data for underlying data. (TIF) [file pbio.3003181.s006.tif]

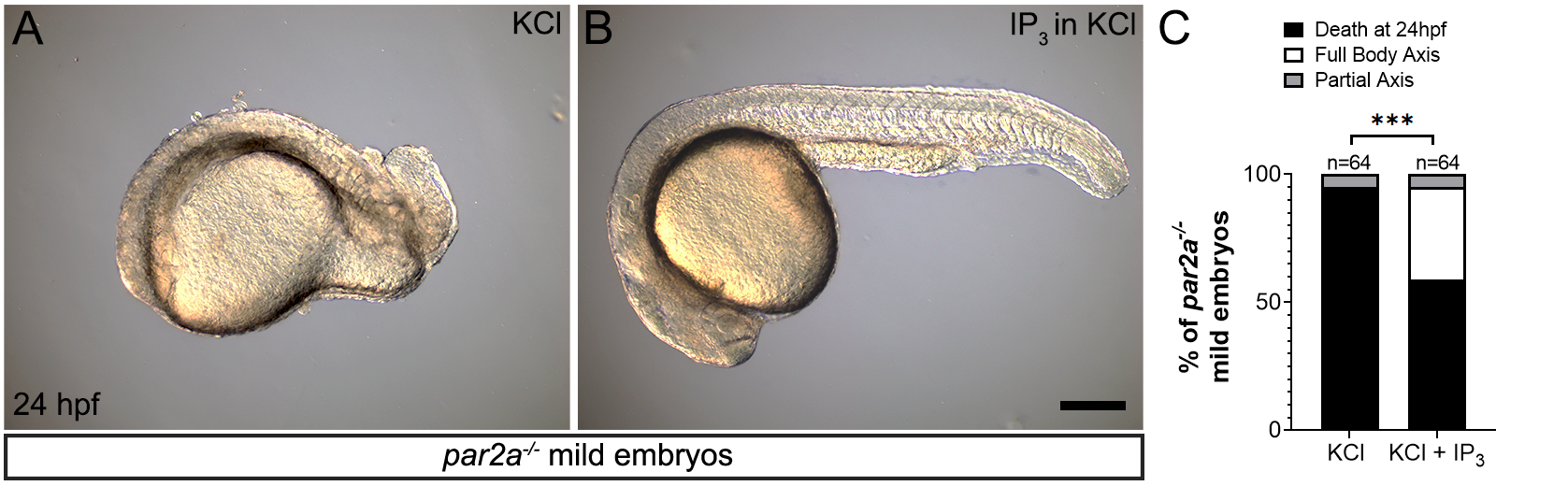

Supplement: S7 Fig — A, B Lateral Nomarski images of naturally fertilized 24 hpf mild par2a embryos injected with either KCl (A) or 20pmol IP3 in KCl (B). C: Proportion of mild par2a−/−- embryos from a single clutch failing to gastrulate or showing full or partial body axis following KCl or IP3 injection. Chi-squared analysis; *** = p < 0.001. Scale bar: B = 200 µm. See file S1 Data for underlying data. (TIF) [file pbio.3003181.s007.tif]

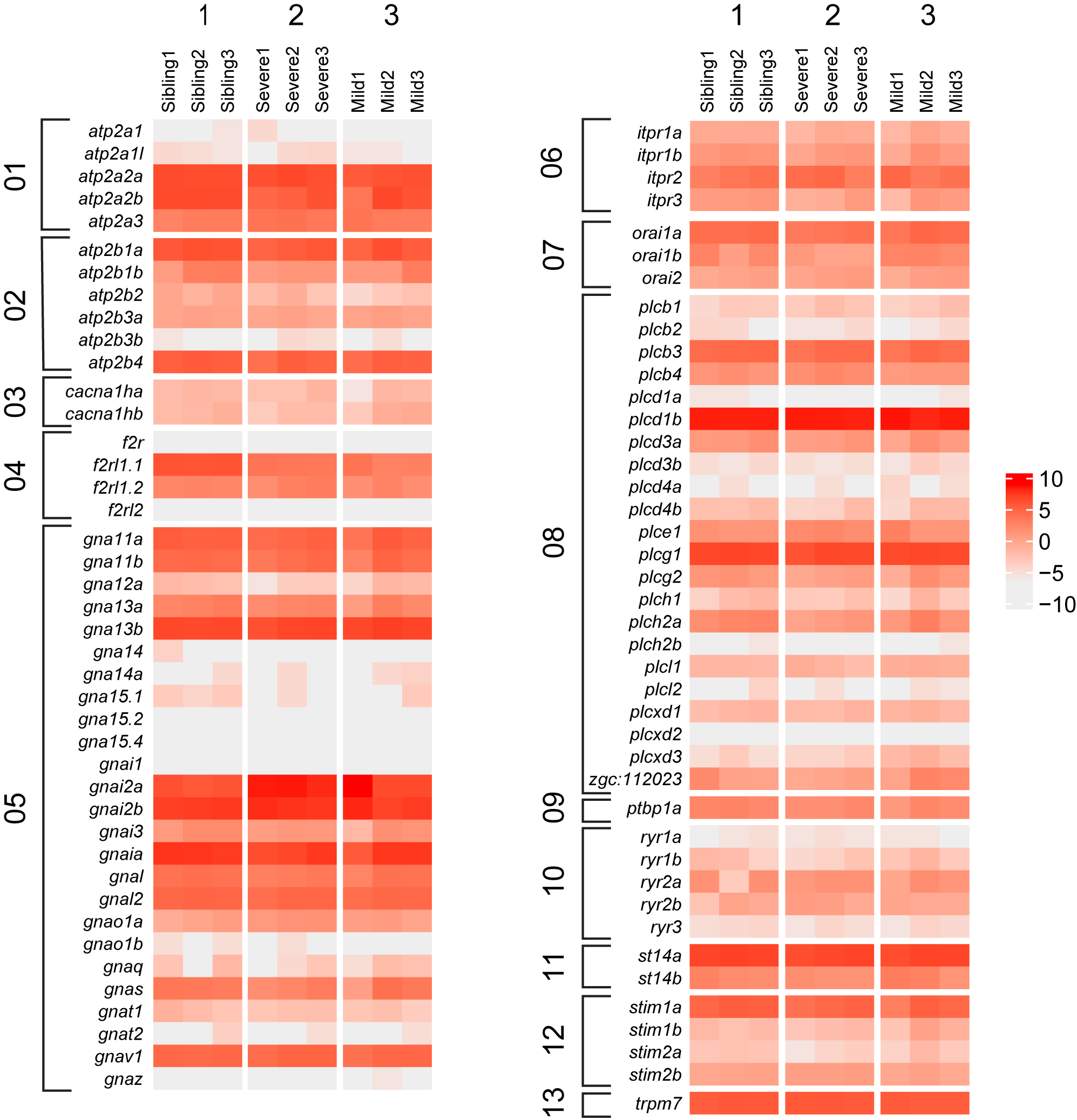

Supplement: S8 Fig — Heatmap of transcript levels of genes involved in calcium regulation in early zygote and blastula stages. Transcript levels were derived by high throughput RNA-seq of mRNA from severe and mild par2alkc4 mutants and sibling zygotes. Genes are grouped by family in rows and triplicate biological replicates are grouped by columns. See file S2 Data for underlying data. (TIF) [file pbio.3003181.s008.tif]

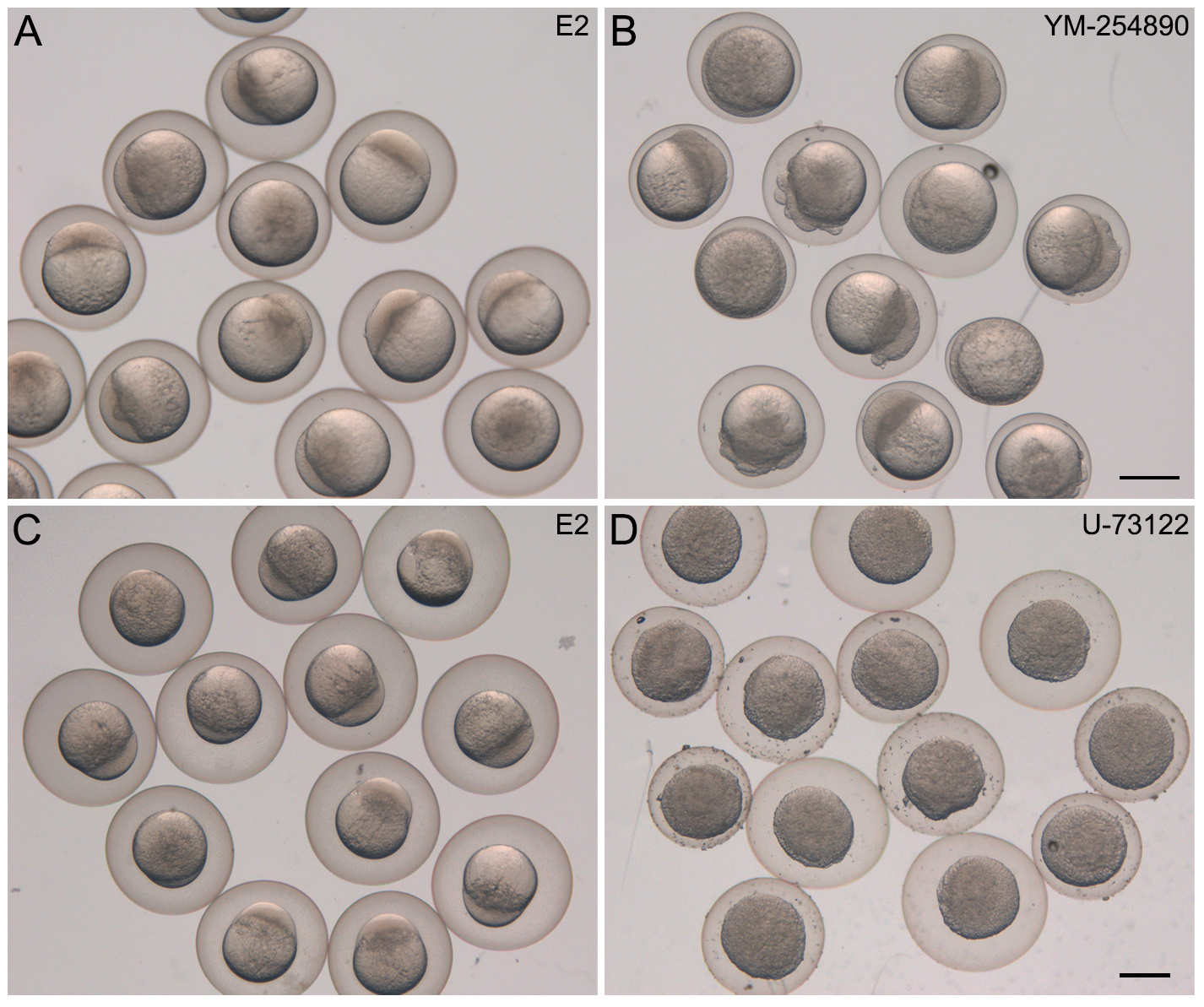

Supplement: S9 Fig — Inhibition of G alpha-q/11 and PLCβ disrupts egg activation. A–D Eggs squeezed from WT females and held in Hank’s buffered saline with BSA. Controls were then activated with E2 medium (A, C). To inhibit G alpha-q/11, eggs were incubated in 200 µM YM-254890 in Hank’s buffer for 20 min then treated with E2 containing 200 µM YM-254890 (B). To inhibit PLCβ, eggs were incubated in 100 µM U-73122 in Hank’s Buffer for 20 min then treated with 100 µM U-73122 in E2 (D). Scale bars: B, D = 500 µm. (TIF) [file pbio.3003181.s009.tif]

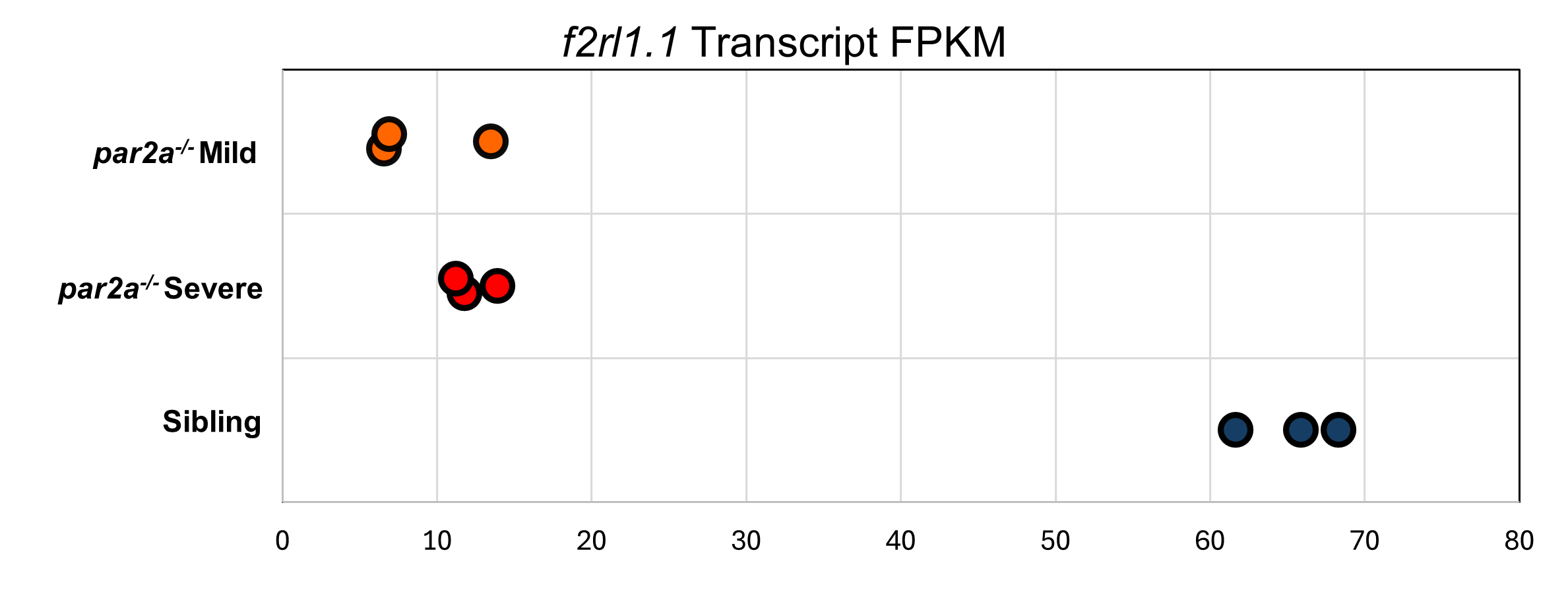

Supplement: S10 Fig — FPKM counts for f2rl1.1 (par2a) transcripts from High throughput RNA sequencing of severe par2a (red circles), mild par2a (orange circles), and sibling zygotes. Each biological replicate shown as a single point. See file S2 Data for underlying data. (TIF) [file pbio.3003181.s010.tif]
