# Supplementary material for: Protease-mediated activation of Par2 elicits calcium waves during zebrafish egg activation and blastomere cleavage
Source: PLoS Biol. 2025 Jun 17;23(6):e3003181. doi: 10.1371/journal.pbio.3003181 (PMC12173237; doi:10.1371/journal.pbio.3003181)
Supplement: S1 Table — (DOCX) [file pbio.3003181.s011.docx]

| **Wild-type ♂ x** | | Reduced Chorion Elevation | Blastodisc Absent | Defective Cell Division | Fails to Gastrulate | Total no. of embryos in clutch |
| --- | --- | --- | --- | --- | --- | --- |
| *par2a^lkc6^* | ♀ 1 | 0% | 0% | 100% | 95% | 70 |
|  | ♀ 2 | 0% | 26% | 74% | 98% | 127 |
|  | ♀ 3 | 48% | 40% | 52% | 100% | 80 |
| *par2a^lkc4^* | ♀ 1 | 4% | 71% | 29% | 100% | 59 |
|  | ♀ 2 | 100% | 100% | 0% | 100% | 48 |
|  | ♀ 3 | 31% | 92% | 1% | 100% | 65 |
| *par2a^lkc5^; par2b^lkc7^* | ♀ 1 | 1% | 14% | 86% | 98% | 244 |
|  | ♀ 2 | 70% | 57% | 40% | 100% | 74 |
|  | ♀ 3 | 0% | 67% | 32% | 100% | 100 |
|  |  |  |  |  |  |  |
| Wild-type ♀ x | |  |  |  |  |  |
| *par2a^lkc4^* | **♂** 1 | 0% | 0% | 0% | 0% | 219 |
|  | **♂** 2 | 0% | 0% | 0.5% | 0.5% | 190 |
|  | **♂** 3 | 0% | 0% | 2% | 2% | 152 |
