# Supplementary material for: Protease-mediated activation of Par2 elicits calcium waves during zebrafish egg activation and blastomere cleavage
Source: PLoS Biol. 2025 Jun 17;23(6):e3003181. doi: 10.1371/journal.pbio.3003181 (PMC12173237; doi:10.1371/journal.pbio.3003181)
Supplement: S2 Table — (DOCX) [file pbio.3003181.s012.docx]

| Wild-type ♂ x | | Clutch number | Reduced Chorion Elevation | Blastodisc Absent | Defective Cell Division | Fails to Gastrulate | Total no. of embryos in clutch |
| --- | --- | --- | --- | --- | --- | --- | --- |
| *par2a^lkc4^* | ♀ 1  (Strong) | 1 | 98% | 88% | 12% | 100% | 116 |
|  |  | 2 | 97% | 84% | 16% | 100% | 151 |
|  |  | 3 | 95% | 92% | 8% | 100% | 150 |
|  | ♀ 2  (Strong) | 1 | 98% | 33% | 65% | 100% | 86 |
|  |  | 2 | 95% | 7% | 93% | 100% | 135 |
|  |  | 3 | 72% | 23% | 76% | 100% | 98 |
|  | ♀ 3  (Mild) | 1 | 4% | 0% | 100% | 100% | 225 |
|  |  | 2 | 4% | 1% | 99% | 100% | 330 |
|  |  | 3 | 10% | 9% | 91% | 100% | 126 |
|  | ♀ 4  (Mild) | 1 | 16% | 1% | 99% | 99% | 119 |
|  |  | 2 | 0% | 0% | 100% | 100% | 61 |
|  |  | 3 | 4% | 2% | 100% | 100% | 49 |
