# Supplementary material for: Protease-mediated activation of Par2 elicits calcium waves during zebrafish egg activation and blastomere cleavage
Source: PLoS Biol. 2025 Jun 17;23(6):e3003181. doi: 10.1371/journal.pbio.3003181 (PMC12173237; doi:10.1371/journal.pbio.3003181)
Supplement: S3 Table — (DOCX) [file pbio.3003181.s013.docx]

|  | Reduced Chorion Elevation | Blastodisc Absent | Blastodisc Deformed | Total no. of embryos in clutch |
| --- | --- | --- | --- | --- |
| Control | 0% | 0% | 0% | 105 |
| 200µM YM-254890 | 11% | 11% | 77% | 158 |
|  |  |  |  |  |
| Control | 2% | 0% | 1% | 94 |
| 100µM U-73122 | 30% | 100% | 0% | 166 |
