# Supplementary material for: Protease-mediated activation of Par2 elicits calcium waves during zebrafish egg activation and blastomere cleavage
Source: PLoS Biol. 2025 Jun 17;23(6):e3003181. doi: 10.1371/journal.pbio.3003181 (PMC12173237; doi:10.1371/journal.pbio.3003181)
Supplement: S4 Table — (DOCX) [file pbio.3003181.s014.docx]

| Gene | Allele | Lesion | Primer name | Primer sequence 5' --> 3' | Method details |
| --- | --- | --- | --- | --- | --- |
| *par2a* | *lkc4* | *8bp del. + 103bp ins.* | par2a-PCR-F | CTGGTCGTGTGGGCTTTAAC | WT band: 132bp Mutant band: 227bp |
|  |  |  | par2a-PCR-R | CAGGAAGTAGATGCAGGGGT |  |
|  |  |  |  |  |  |
|  | *lkc5* | *8bp del.* | **par2a-seqF** | **TCCACAGCTTTCATCGCATG** | PCR then use **par2a-seqF** primer for sequencing |
|  |  |  | par2a-PCR-R | CAGGAAGTAGATGCAGGGGT |  |
|  |  |  |  |  |  |
|  | *lkc6* | *1bp del.* | lkc6-F-WT | ATGGATATAGTCACCTGCCAT | Run PCR for WT allele with lkc6-F-WT and lkc6-R-common primers Run PCR for Mutant allele with lkc6-F-mut and lkc6-R-common primers |
|  |  |  | lkc6-F-mut | ATGGATATAGTCACCTGCCAA |  |
|  |  |  | lkc6-R-common | GCATCTGCACGTTTGACACT |  |
|  |  |  |  |  |  |
| *par2b* | *lkc7* | *2bp del. + 1bp ins.* | par2b-seq-F | ACCCAGCTGCCATTTACATG | PCR then use **par2b-seq-R** primer for sequencing |
|  |  |  | **par2b-seq-R** | **GCACCGTGACGATGAGAATC** |  |
