# Supplementary material for: Protease-mediated activation of Par2 elicits calcium waves during zebrafish egg activation and blastomere cleavage
Source: PLoS Biol. 2025 Jun 17;23(6):e3003181. doi: 10.1371/journal.pbio.3003181 (PMC12173237; doi:10.1371/journal.pbio.3003181)
Supplement: S5 Table — (DOCX) [file pbio.3003181.s015.docx]

| Gene | Primer name | Primer sequence 5' --> 3' |
| --- | --- | --- |
| *par2a* | par2a-F-TC | TAATGGCGCACGGACTGATC |
|  | par2a-R-TC | CGATGATGTAGAGCACGGGG |
|  |  |  |
| *eef1a1l1* | qPCR-ef1a-F | CTGGAGGCCAGCTCAAACAT |
|  | qPCR-ef1a-R | ATCAAGAAGAGTAGTACCGCTAGCATTAC |
