# Supplementary material for: Protease-mediated activation of Par2 elicits calcium waves during zebrafish egg activation and blastomere cleavage
Source: PLoS Biol. 2025 Jun 17;23(6):e3003181. doi: 10.1371/journal.pbio.3003181 (PMC12173237; doi:10.1371/journal.pbio.3003181)
Supplement: Attachment — Submitted filename: Response to reviewers.pdf [file pbio.3003181.s030.pdf]

	Reviewer1	
1.1	That is which Galpha and/or PLC are involved.	We used RNA-seq to determine the isoforms expressed in early Wild-Type zygotes, noting that *gna11a*, *gna11b* and *plcb3* are the predominating Gq and PLCβ expressed genes, however there is strong expression of *gna13b,* and *gnai* orthologues. This is presented as a heatmap in a new Supplemental Figure and also as a new Supplemental Data File. We have used Gq/11 and PLC inhibitors to show that these are required for normal egg activation, adding a new supplementary figure and table. There is extensive literature that Par2 acts via Gq/11 and PLCβ. We have added reference to this.
1.2	It would also be important to test the expression levels of PLCs, Galphas, IP3 receptor isoforms, and other Ca2+ transporters in the par2 mutants as compared to WT as this may help define the differential phenotype in some par2 mutant females	To do this we performed bulk RNA-seq on very early mild, severe and sibling *par2a* mutant zygotes to compare mRNA levels of the main defined Ca^{++} regulators in eggs. There was no clear candidate that leaps out. o*rai1b* and a *plcxd1* paralogue are slightly upregulated but it is hard to see that this would be sufficient to account for the differential expressivity. We were disappointed that this approach did not enlighten us, but we have presented the results in a new Supplemental Figure and also as a new Supplemental Data File for anyone in the field to analyse. We have submitted all raw files to NCBI GEO.
1.3	A body of work describing the differentiation of Ca2+ signaling in preparation for fertilization in xenopus is quite relevant to the introduction and needs to be cited (Machaca, 2007; Nader et al., 2013)	These have been added to the introduction.
1.4	Need to show Western blots from eggs confirming the generation of the mutants par2 proteins and their penetrance at the protein level in eggs.	Unfortunately, there is no antibody cross reactive with zebrafish Par2a protein. However, we performed qPCR to determine if the *par2a* mutant transcripts were subject to nonsense mediated decay, finding that they were. The mutants had an approximately 80% reduction in transcript levels. We have added this to the results section and a new supplementary figure. The reduction in *par2a* mutant transcript levels was also confirmed in our RNA-seq data comparing transcriptomes of sibling and maternal mutant zygotes. This decay suggests the levels of mutant protein are likely to be lower in addition to lacking function.
1.5	Also some measure of the fertility rates of female par2-/- needs to be assessed to confirm that it correlates with the observed phenotypes at the egg level.	No par2a mutant female is fertile. All eggs derived from these mutants by natural crosses fail to gastrulate successfully and most either lack a blastodisc or have such disrupted cell division that they abort before the start of gastrulation. Fecundity, measured by clutch size, does not appear affected, however. Clutch size is highly variable in zebrafish and depends on many factors such as tank density and age. There was no significant difference in clutch size between sibling (Ave. 251 eggs), mild mutant (Ave. 201 eggs), and severe mutant (Ave.

		326 eggs) females measured across 6 clutches each. We have added comment on this to the results.
1.6	Male fertility for these mutants also needs to be assessed to support the conclusion that the phenotype is egg specific.	Mutant males have not shown a fertility defect in any allele, and indeed we use them to propagate the alleles by crossing to heterozygous females. We have added data to Supplementary Table 1 for 3 mutant males showing all are fertile and referred to this in the results section.
1.7	I am confused a little here as the authors state that par2 mutants never gastrulate which implies that homozygous par-/- females should not produce viable offspring? Yet they also state that par2 mutant animals reach adulthood normally? Please clarify.	This is a function of the genetics of the oocyte/egg prior to zygotic genome activation. As the genome does not activate until around 3 hours after fertilisation, early events of the egg/zygote rely on proteins and RNA that have been deposited by the mother during oogenesis. Thus, for very early events of development prior to 3hpf, the genotype of the mother is relevant, not the genotype of the zygote. Thus, a *par2a* homozygote embryo derived from a heterozygote mother (Z*par2a*$^{-/-}$) will not have an egg activation phenotype, whilst a heterozygous embryo derived from a homozygous mother (M*par2a*$^{+/-}$) will. We can thus raise *par2a*$^{-/-}$ mutant fish only if they are derived from heterozygous mothers. We have clarified in that statement about mutants failing to gastrulate that this refers to maternal mutants.
1.8	The differential phenotype severity in par2 mutant females is attributed to 'differential expressivity'. As the females are homozygous for a non-functional par2 mutant this differential expressivity is likely due to another pathway partially compensating. Some discussion of what those could be would be useful as par2b is ruled out.	We tried to identify this by comparative RNA-seq between mild and severe *par2a* mutants, however no strong candidate emerged. We have included the details in the revised manuscript. We have also expanded the section in the discussion about possible pathways might be compensating, in particular the Src family kinases as these have been linked to PLCγ activity in xenopus and zebrafish.
1.9	Fig. 4: treating embryos with ionomycin to increase intracellular Ca2+ as mentioned is non-specific and does not appropriately maintain the spatial and temporal dynamics of the physiological Ca2+ signal at the cleavage furrow. A more elegant and better controlled experiment, if technically doable, would be to inject one of the two blastomeres with IP3 and assess division as the authors are postulating a par2 depend Ca2+ release mechanism IP3 inject should have a more specific rescue effect.	This was a neat idea and we tried it, but it was not easy to do and the results were difficult to interpret. Functional gap junctions are widely expressed in the early blastomeres (Kimmel CB, Law RD. Developmental Biology. 1985;108(1):78-85) so this would have likely rapidly homogenised levels of IP3 between blastomeres following injection.

1.10	Fig. S5: 2APB is not a specific IP3R inhibitor and blocks among other Ca2+ signalling proteins store-operated Ca2+ channels. As such it cannot be used to conclude that IP3R are involved. A more specific blocker such as xestospongin is needed.	We tried this at one concentration however this did not affect egg activation and we did not have sufficient amounts to try at a higher concentration. We are really sorry this didn't work but think that earlier treatments need higher concentration than later treatments (this was true for 2-APB). We found further corroborating literature on the use of independent IP3Rs inhibitors in zebrafish including IRBIT and Heparin, resulting in similar effects to 2-APB, and have now included these in the results section for the reader. This is also consistent with many cited precedents set in other organisms on the effect of IP3R inhibition on cell division and egg activation.
	**Reviewer 2**	
2.1	How many independent par2a alleles were evaluated in par2a-/- eggs? The text reports three (second paragraph of "Maternal par2a mutants..." section), but there are only 2 reported in Supp Table 1, with the 3rd allele (lkc5) presumably generated in a par2b background, according to the methods	Yes, you are correct. We first generated and analysed two *par2a* alleles (*lkc4* and *lkc6*). When it became clear that there was variability in the phenotype, we decided to make the double mutants with *par2b* in case it was functionally compensating to a variable extent. We generated the third *par2a* allele (*lkc5*) in the *par2b* mutant background. We saw no difference in phenotype or variability, thus, indicating that *par2b* did not compensate for *par2a* in this context. We have updated the text to be explicit that we assessed two single alleles, and we have expanded the text on how we generated the double mutant by sequential CRISPR mutagenesis of *par2b* and *par2a*.
2.2	To what extent were multiple clutches from the same par2a-/- female evaluated for consistent degree of expressivity? This is reported in passing, but no data are shown (Supp Table 1 seems to report numbers for only a single clutch per female)	We did this often in the course of our work and they were highly consistent in severity. We have now added a new Supplementary Table 2 comparing the phenotype severity in 3 clutches from each of 4 mutant females (both mild and severe) showing the consistency of expressivity.
2.3	What is the relationship between the par2a-/- females of the same allele? Are they siblings? Or were there multiple different parents?	We have analysed both. In Supplementary Table 1 they were siblings. We have updated the Table title to indicate this. We have also looked across different generations and seen the same variability in phenotype based on the mother and not the allele. We have not yet seen any drift towards one extreme of severity. We have expanded the description of this in the results section.
2.4	The methods suggest that F0 founders may have been incrossed to potentially yield homozygous (or rather, transheterozygote/heteroallelic) F1. Can the authors clarify the generations and the genotyping that yielded the final par2a-/- females whose eggs were evaluated?	We incrossed potential founders, but as the mutation rate was rather low, only 1 founder in a cross ever had a mutation, so they were essentially outcrosses. We then incrossed each allele. Thus, we never made transheterozygotes. I altered the text in the methods section to specify this better.
2.5	What primers were used for genotyping / sequencing?	All genotyping primers and methods are presented in a new Supplementary Table 4

2.6	The large insertion in the lkc4 allele appears to be cas9 sequence, suggesting that cas9 DNA was available in the cell to serve as a repair template... The lkc7 par2b allele is also quite unusual given how far the lesion is from the PAM site. If there are any additional methodological details that should be documented, I encourage the authors to do so	We didn't realise this! We originally BLASTed against the zebrafish genome only and never considered it might be Cas9 sequence. We suspect strongly that it is a hangover from the Cas9 template plasmid. In fact, speaking to a colleague, he has seen T7 promoter sequence knock into CRISPR sites before, so there is precedent (Christian Mosimann personal communication). I have included some more details of our sgRNA and Cas9 RNA synthesis and mentioned this homology in the figure legend of Supplementary Figure 2.<br><br>For the *lkc7* allele, I have to confess this was my error as I did not realise that Jiajia used a new guide RNA to generate this allele, and I assumed she used an older one we had originally developed. In hindsight I should have checked when this allele location looked so distant from the gRNA binding site. I have now updated this figure and Materials and Methods with the correct guide RNA sequence and location. I do apologise for my confusion on this.
2.7	In the intro and results (last paragraph of "Maternal par2a mutants...") the authors state that egg activation in fish is triggered by spawning/water; however, this is not the case for all fish -- notably medaka eggs do not activate in water but rather coupled with fertilization (Iwamatsu 1991)	This is a good and important point, and we have corrected these statements and added a comment in the discussion about the difference egg activation timing in medaka with the Iwamatsu reference. Thank you for pointing this out.
2.8	par2a/b have updated gene symbols, f2rl1.1 and f2rl1.2 respectively, so the authors should report these as well as the relevant accession numbers (Refseq? Ensembl?) that they based their gene models on	Good idea. We clarified in the introduction that Par2 is encoded by the *F2rl1* gene, added the relevant zebrafish synonyms to the results section and again in the materials and methods section with the relevant Gene ID from RefSeq, NCBI.
2.9	The authors may want to note that par2b seems to be only weakly maternal in contrast to par2a according to RNA-seq datasets (e.g., White et al 2017), which may account for its lack of involvement in egg activation	Good point. We have identified this also through our RNA-seq analysis and included this in our supplementary data as well as presenting this in the discussion. Further, our comparative RNA-seq analysis showed there was no upregulation of *par2b* in *par2a* mutants ruling out a genetic compensation mechanism (which was possible in the mutants). Finally, as the expressivity shows the behaviour of and independently assorting trait, it cannot be linked to the *par2a* lesion, also arguing against *par2b* compensating for *par2a* in egg activation. We have also added a comment on this to the discussion.
2.10	matriptase1 (st14a/b) is also maternal, perhaps relevant to the discussion in speculating	We do have data on this. We have previously made the maternal zygotic *st14a* and maternal zygotic *st14a;st14b* double mutants for our previous paper. Neither showed an egg activation phenotype, but solely an ear otolith

	what the maternal protease may be	phenotype. We have included this in the discussion with reference to our previous paper and also to our new RNA-seq data (Supp Fig 8 and Supp File 1) demonstrating early expression in zygotes.
2.11	par2a and par2b are tandem duplicates very close to each other on the chromosome, which has implications for the genetics (i.e., mutating one locus may have a regulatory consequence on the other); but also an intriguing possibility for functional divergence due to duplication, given that Par2b's role in inflammation may be more conserved	Others have shown that while both Par2a and Par2b are both expressed maternally in zebrafish development, only Par2b is expressed in late embryogenesis. In the adult both seem to be expressed again. This does indeed indicate that there is likely sub-functionalisation and functional divergence from the ancestral state.
2.12	In the GCaMP fluorescence figures, please indicate in the legend that the images are pseudocolored and what the intensity values represent (normalized? ratio?)	These are normalised and pseudo-coloured using the “16 color” Lookup Table of ImageJ. We have added these details to the materials and methods ion the GCaMP6 imaging section and also to all figure legends in the main and supplementary figures.
2.13	Fig 3D, these are animal view? Indicate this in the legend	We have added that they are lightsheet images of the animal pole in both D and E.
2.14	In Supp Fig 2a/b, the positions of the blue bars are off compared to the sequences of the gRNAs reported (they are too short and slightly shifted)	We have corrected this and included the PAM site for clarity.
	**Reviewer 3**	
3.1	The timing of the Ca2+ rises could be better presented. In all figures, the concentrations of Ca2+ seem to be rising from the outset. Could the authors inhibit fertilization with BSA and then wash it off while monitoring Ca2+? In this manner, the Ca2+ rise can follow an established baseline.	This is in fact what we did. The problem was that the rise is so rapid (within seconds) that by the time we refocussed the microscope, the movie started 30sec after exposure to the medium. We are sorry we were not able to do this faster. But we still think our movies demonstrate a reduced duration and amplitude of calcium release in out mutants and treatments.
3.2	The authors could demonstrate whether the timing of Ca2+ release and egg activation are the same and whether induced by spawning or by spawning + fertilization. In other words, is the Ca2+ signal modified at all by the presence of fertilization?	There is conflicting literature on this. Originally Lee et al (1999) concluded that calcium wave propagation is identical in extent and speed in eggs activated with and without sperm. Sharma and Kinsey (2008) suggested sperm generates a short rapid Ca^{++} wave (30sec post fertilisation) at the cortex, which is slower (at 10 minutes) in parthenogenetically activated eggs. We repeated this comparison and observed identical rapid calcium waves initiating about 30sec after both fertilisation and parthenogenetic activation, supporting Lee et al’s original conclusion. We have added this as a new Supplementary Movie 1.

3.3	Similarly, how the protease receptor is activated is not known. Is there a protease in the CGs? This is something they propose. The authors could block CG exocytosis or reduce it using pharmacological inhibitors (Myosin II inhibitors) and examine if there is any difference in egg activation. This would get close to answering whether the protease is a component of the cortical granules. If it is not affected, it could be that this receptor is constitutively active, and the simple dilution effect may activate it. Have the authors used different ratios of eggs to media to see if this affects the rate of activation and Ca2+ elevation?	We tried using phalloidin to crosslink actin as this has been shown to block cortical granule release in zebrafish (Becker, K. A., & Hart, N. H. (1999). *Journal of Cell Science*, *112* 97–110.). We needed to permeabilise the egg prior to treatment but permeabilization alone deformed the embryos significantly, making it difficult to interpret effects of phalloidin. We have removed reference to the protease being in cortical granules as this was entirely speculative on our part and simply suggested the protease could be sequestered. Calcium assays performed by Kim et al (Kim S, Carrillo M, Kulkarni V, Jagadeeswaran P (2009) PLOS ONE 4(12): e8403.) indicates that zebrafish Par2a is not constitutively active. We compared activation of 100 eggs in 500µl E2 medium vs 40 eggs in 20000µl E2 but saw no difference in activation rate, so although there may be a dilution requirement, dilution is likely to be potent in effect. We believe that given the potent effects of exogenous serine protease inhibitors on blocking activation, and the presence of serine proteases in fish ovarian fluid, that the dilution mechanism is likely, but that other methods are quite possible.
3.4	The authors should address the issue of why the ionophore exposure rescues egg activation but not cleavage, whereas IP3 does both. Does IP3 persist from the initial bolus injection and support those initial cleavages? The failure of Ionomycin would suggest that the internal PLC is not sensitive to Ca2+.	We believe that this is due to the highly localised distribution of ER and IP_3R at the cleavage furrow as previously demonstrated by Lee et al (2003). Thus, IP_3 injection can still generate localised Ca^{++} release at the furrow for proper cytokinesis, as its receptor is spatially restricted. Use of an ionophore however would elevate Ca^{++} globally throughout the cell, aborting Ca^{++} localisation, and thus disrupting normal cleavage. We have added this to the discussion.
3.5	What about the location and expression of the putative receptor? Is there an antibody to demonstrate expression and distribution?	There is no antibody that cross reacts to fish Par2a, however we made an eGFP fusion construct and made a transgenic line expressing this under a maternal oocyte promoter, *zp3*. This allowed us to image the dynamics of Par2a-eGFP during early cleavages. We present this timelapse as a new Supplementary Movie 9.
3.6	Can the expression of the receptor into the Par mutants rescue the phenotype? This will be important and, equally, the receptor's expression levels could be titrated.	This is technically very challenging. We cannot rescue with RNA injection as it needs time to be translated, and the phenotype occurs extremely early. We enquired about getting the protein synthesised but 7-pass transmembrane proteins are difficult to express with in vitro systems and may not fold properly. Our protein production unit here couldn't do it. The only way we can think of doing this is to make a transgenic line that expresses in ovaries and cross it to the mutants. This would take at least 3 generations if the overexpression itself wasn't detrimental. We tried to see if our fusion Par2a-eGFP (see above) would rescue, but it couldn't, and we suspect the C-terminal eGFP is disrupting signalling somehow.

3.7	In the introduction, a better explanation of when fertilization occurs in the context of egg activation will be beneficial. If egg activation is independent of fertilization, does it mean both occur simultaneously or nearly so to avoid engaging the polyspermy block?	Yes, this is correct. Release of eggs is induced during courtship by the male. We have expanded the second paragraph of the introduction to better explain zebrafish natural mating and how egg activation and fertilisation occur as separate, parallel processes.
3.8	The discussion needs a completed final sentence. As it ends now, it seems incomplete	We've added a concluding sentence contextualising the relevance of the work to the field.
3.8	It is unclear the connection to SOCE in the following statement, "Par2a as an essential regulator of IP3 levels at the zebrafish blastomere cleavage furrow, and acts with a known role of Store Operated Calcium Entry to replenish ER Ca++ levels (21)." The statement needs to be better explained, as SOCE was not mentioned previously, and the evidence in fish was not discussed.	We have added statements to both the introduction and to the discussion outlining the work from the Miller and Webb labs on localisation and inhibition of SOCE components during blastomere furrow deepening. We have added reference to the localisation paper.
